# The effect of 3D carbon nanoadditives on lithium hydroxide monohydrate based composite materials for highly efficient low temperature thermochemical heat storage

**DOI:** 10.1039/c8ra00269j

**Published:** 2018-02-21

**Authors:** Shijie Li, Hongyu Huang, Jun Li, Noriyuki Kobayashi, Yugo Osaka, Zhaohong He, Haoran Yuan

**Affiliations:** Key Laboratory of Renewable Energy, Guangdong Provincial Key Laboratory of New and Renewable Energy Research and Development, Guangzhou Institute of Energy Conversion, Chinese Academy of Sciences No. 2 Nengyuan Rd, Wushan, Tianhe District Guangzhou 510640 P. R. China lisj@ms.giec.ac.cn huanghy@ms.giec.ac.cn hezh@ms.giec.ac.cn yuanhr@ms.giec.ac.cn; University of Chinese Academy of Sciences Beijing 100049 PR China; Nagoya University Furo-cho, Chikusa-ku Nagoya-shi Aichi 464-8603 Japan Junli@energy.gr.jp kobayashi@energy.gr.jp; Kanazawa University Kanazawa-shi Ishikawa-ken 920-1192 Japan y-osaka@se.kanazawa-u.ac.jp

## Abstract

Lithium hydroxide monohydrate based thermochemical heat storage materials were modified with *in situ* formed 3D-nickel-carbon nanotubes (Ni-CNTs). The nanoscale (5–15 nm) LiOH·H_2_O particles were well dispersed in the composite formed with Ni-CNTs. These composite materials exhibited improved heat storage capacity, thermal conductivity, and hydration rate owing to hydrogen bonding between H_2_O and hydrophilic groups on the surface of Ni-CNTs, as concluded from combined results of *in situ* DRIFT spectroscopy and heat storage performance test. The introduction of 3D-carbon nanomaterials leads to a considerable decrease in the activation energy for the thermochemical reaction process. This phenomenon is probably due to Ni-CNTs providing an efficient hydrophilic reaction interface and exhibiting a surface effect on the hydration reaction. Among the thermochemical materials, Ni-CNTs–LiOH·H_2_O-1 showed the lowest activation energy (23.3 kJ mol^−1^), the highest thermal conductivity (3.78 W m^−1^ K^−1^) and the highest heat storage density (3935 kJ kg^−1^), which is 5.9 times higher than that of pure lithium hydroxide after the same hydration time. The heat storage density and the thermal conductivity of Ni-CNTs–LiOH·H_2_O are much higher than 1D MWCNTs and 2D graphene oxide modified LiOH·H_2_O. The selection of 3D carbon nanoadditives that formed part of the chemical heat storage materials is a very efficient way to enhance comprehensive performance of heat storage activity components.

## Abstract 2

3D carbon modified LiOH·H_2_O particles were well dispersed into nanoparticles (5–15 nm) and tested using *in situ* DRIFT spectroscopy.

## Graphical abstract

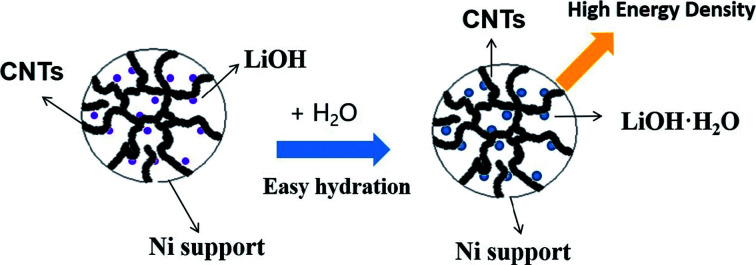

## 1. Introduction

As an important part of clean and efficient utilization of alternative energy, the development of thermal energy storage technology has become increasingly important in recent years owing to the incremental consumption of fossil energy and the emission of greenhouse gases.^1,2^ These technologies are divided into three main types: sensible heat storage,^3,4^ latent heat storage,^5,6^ and thermochemical heat storage.^7,8^ All of these technologies participate in solving the supply and demand mismatch of thermal energy, and improve energy efficiency.^9^ Among these technologies, thermochemical heat storage, which uses reversible chemical reactions to store and release thermal energy, facilitates the efficient utilization of thermal energy due to its high heat storage density.^10^ Based on heat storage working temperature, the thermochemical heat storage technology could be divided into two parts: high temperature heat storage and low temperature heat storage.^9,10^ As core components of these technologies, numerous thermochemical materials (TCMs) have been selected. For instance, metal hydroxides, metal hydrides, and metal carbonates are typically used as TCMs for high-temperature thermochemical heat storage, whereas inorganic salt hydrates and salt ammoniate are considered as promising candidates for low-temperature thermochemical heat storage due to their different decomposition temperature.^11–16^ For efficiently storing low-temperature thermal energy, the inorganic hydrate lithium hydroxide monohydrate (LiOH·H_2_O), which possesses high energy density (1440 kJ kg^−1^) and mild reaction process, was selected as a potential material.^17^ However, similar to other inorganic hydrates^18,19^ pure LiOH·H_2_O exhibit low hydration rate and thermal conductivity;^15,17^ moreover, its heat storage density decreases after hydration, thereby seriously limiting the application of the material. Hence, composite TCMs with high heat storage density and high thermal conductivity are of considerable synthetic value.

3D carbon nanomaterials (3D-carbon nanotube sponge and arrays^20,21^), which exhibit large surface area, low bulk density, and chemical stability,^22–24^ are widely used in various fields, such as electronics^25,26^ and catalysis^24,27^ as typical carbon nanomaterials. In addition, these materials offer excellent hydrophilic property after the introduction of surface oxygen groups. However, under normal conditions, traditional one-dimensional or two-dimensional carbon nanomaterials are selected and used for latent heat thermal energy storage.^2,28,29^ 3D carbon nanomaterials are rarely used for thermal energy storage,^30^ especially thermochemical heat storage. In our previous work, 1D carbon nanomaterials,^31^ MWCNTs were used to modify LiOH·H_2_O; heat storage performance was markedly enhanced, but the thermal conductivity needed improvement. Hence, 2D-carbon nanomaterial^15,32^ graphene oxide was selected and, which markedly improved the energy storage performance of LiOH·H_2_O and Mg(OH)_2_. Graphene oxide also positively affected thermal conductivity to a certain extent. Hydrophilic substances, such as lithium chloride (LiCl), 13X-zeolite, and NaY-zeolite, were also used to enhance the heat storage performance and obtain expected results.^33^ Kato *et al.* investigated the reaction behavior of metal-salt modified Mg(OH)_2_-based materials; during the heat storage process, LiCl and LiBr acted as catalysts and decreased the activation energy effectively and substantially improved heat storage performance.^5,11,34^ Moreover, the functional groups on the surface of the materials markedly affected the reaction behavior of Mg(OH)_2_. However, the relationship between heat storage density and particle size, especially when materials reached nanoscale, was not discussed. In this work, *in situ* DRIFT spectroscopy technology and chemical reaction kinetics test were used for an in-depth analysis of the heat storage mechanism, which has not been involved in previous research. Moreover, to simultaneously improve the performance of heat storage density, hydration rate, and thermal conductivity, we synthesized a novel TCM composite of *in situ* formed 3D-nickel-carbon nanotubes (Ni-CNTs) and LiOH·H_2_O. Four kinds of TCMs were prepared, and the effect of 3D-carbon nanomaterials was extensively investigated. The highest heat storage density of Ni-CNTs–LiOH·H_2_O could reach 3935 kJ kg^−1^, which is 2.2 times higher than that of 1D MWCNTs modified LiOH·H_2_O (1804 kJ kg^−1^) and the thermal conductivity (3.78 W m^−1^ K^−1^) is also much higher than MWCNTs modified LiOH·H_2_O (1.75 W m^−1^ K^−1^)^31^ The heat storage density and the thermal conductivity of Ni-CNTs modified LiOH·H_2_O are also higher than 2D graphene oxide modified LiOH·H_2_O (1980 kJ kg^−1^; 1.70 W m^−1^ K^−1^), respectively.^15^ It indicated that the selection of 3D nano carbon materials as composed part of the chemical heat storage materials is a very efficient way to enhance comprehensive performance of heat storage activity component.

## 2. Experimental

### 2.1. Raw materials and synthesis method of LiOH·H_2_O-based TCMs

The 3D-carbon nanotubes were synthesized by catalytic chemical vapor deposition method with C_2_H_4_ as carbon source and nickel foam as catalyst.^35^ First, nickel foam was placed in a tubular furnace, which was heated to 700 °C, and filled with Ar/H_2_ (300 mL/100 mL) mixed reducing gas for 2 h. After reduction, Ar/H_2_ mixed gas was replaced with Ar/C_2_H_4_ (400 mL/100 mL), the temperature was increased to 550 °C, and the system was allowed to react for 20 min. After the temperature decreased to 25 °C, the 3D Ni skeleton CNTs (Ni-CNTs) was obtained. Then, after being oxidized by 5% O_2_ for 2 h at 250 °C, the as-prepared 3D Ni-CNTs were composited with LiOH·H_2_O by impregnation method. First, 0.5 g LiOH·H_2_O was dissolved in 1 mL deionized water under vigorous stirring. Subsequently, 3D nanocarbon were added in the LiOH aqueous solution at room temperature, and the mixture was stirred continuously for 4 h. Afterward, the products were withdrawn and vacuum freeze-dried. The obtained materials with different LiOH·H_2_O content (14 wt%, 23 wt%, 39 wt%, 100 wt%) were named as Ni-CNTs–LiOH·H_2_O-1, Ni-CNTs–LiOH·H_2_O-2, Ni-CNTs–LiOH·H_2_O-3, and LiOH·H_2_O, respectively.

### 2.2. The characterization and heat storage performance test method of LiOH·H_2_O-based TCMs

Surface topography was measured by field-emission scanning electron microscopy (SEM, S-4800, Hitachi Limited). Transmission electron micrographs (TEM) were obtained with FEI Tecnai G212 operated at 100 kV and a JEOL JEM-2100F operated at 200 kV. X-ray diffraction (XRD) analysis was performed on a D8-advance X-ray diffractometer (Bruker, Germany) with Cu target (40 kV, 40 mA). The scan step size was 0.0167°, and counting time was 10.160 s. Nitrogen adsorption–desorption was measured at the boiling point of nitrogen (77 K) by a Quantachrome QDS-30 analyzer. BET (Brunauer–Emmett–Teller) surface area and pore structure were measured by nitrogen physisorption under normal relative pressure of 0.1–1.0. The thermal conductivity of the sample was measured by a DRL-II thermal conductivity tester (Xiangtan Xiangyi Instrument Co., Ltd.). LiOH·H_2_O, Ni-CNTs–LiOH·H_2_O-1, Ni-CNTs–LiOH·H_2_O-2, Ni-CNTs–LiOH·H_2_O-3 were used as raw substance then, LiOH Ni-CNTs–LiOH-1, Ni-CNTs–LiOH-2, and Ni-CNTs–LiOH-3 were synthesized by decomposing LiOH·H_2_O, Ni-CNTs–LiOH·H_2_O-1, Ni-CNTs–LiOH·H_2_O-2, and Ni-CNTs–LiOH·H_2_O-3 in a horizontal tubular quartz furnace with Ar gas at 150 °C for 3 h. Dehydrated products were cooled to 30 °C in an Ar atmosphere, and water vapor at a partial pressure of 2.97 kPa and carried by N_2_ flow was introduced to the tube for 60 min for hydration operation at 30 °C. After hydration, the endothermic heat and temperature of the samples were measured through Thermogravimetry and Differential Scanning Calorimetry (TG-DSC) (STA-200, Nanjingdazhan Co., Ltd.), which was also used for measuring weight change during dehydration step. Each TG-DSC measurement was repeated three times in order to ensure correctness. The activation energy of dehydration performance of all samples was calculated using Ozawa method,^36^ which is applicable for calculating the activation energy of thermal decomposition and dehydration reaction. By using the Ozawa method, the following equation can be obtained based on the reaction rate expression and the Arrhenius's equation:
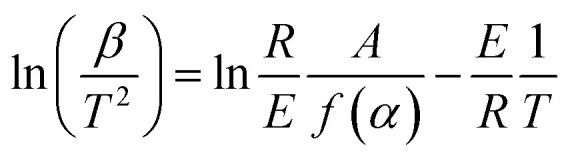


In this equation, *E* is the activation energy (kJ mol^−1^), *β* is the heating rate (K s^−1^), *T* is temperature (K), *R* is the ideal gas constant (J (mol K)^−1^), *A* is a pre-exponential factor, *α* is the dehydration conversion, and *f*(*α*) is a function of dehydration conversion. During the calculation of activation energy, the dehydration conversion was maintained at 70%. The heating rates were 3 K min^−1^, 7 K min^−1^ and 10 K min^−1^, and the activation energy was obtained from the slope (−*E*/*R*) of this equation.

## 3. Results and discussion

### 3.1. Microstructure characterization of LiOH·H_2_O-based TCMs

**Fig. 1:**
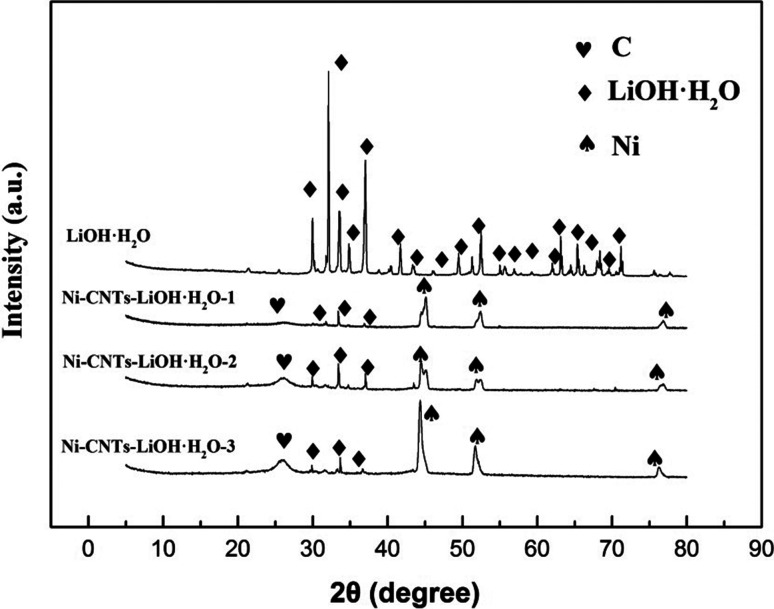
XRD patterns of LiOH·H_2_O, Ni-CNTs–LiOH·H_2_O-1, Ni-CNTs–LiOH·H_2_O-2, Ni-CNTs–LiOH·H_2_O-3.

Fig. 1 shows the XRD patterns of LiOH·H_2_O, Ni-CNTs–LiOH·H_2_O-1, Ni-CNTs–LiOH·H_2_O-2, and Ni-CNTs–LiOH·H_2_O-3 samples. As shown in Fig. 1, the diffraction peaks at around 30°, 32.19°, 33.64°, 34.84°, 37.07°, 38.83°, 40.06°, 41.61°, 43.49°, 49.37°, 51.36°, 52.47°, 55.15°, 55.70°, 56.92°, 62.15°, 63.13°, 64.55°, 65.47°, 66.22°, 68.35° and 71.34°, respectively, were attributed to LiOH·H_2_O, whereas the diffraction peaks at around 25° were attributed to graphitic carbon.^37^ Meanwhile, the diffraction peaks at around 45°, 52°, and 77° could be assigned to metal nickel. It could be clearly seen that the diffraction peaks of LiOH·H_2_O were sharp and strong in the LiOH·H_2_O sample, but the diffraction peaks of LiOH·H_2_O became weaker and more diffused when 3D carbon nanomaterial Ni-CNTs were added. Hence, the composites of Ni-CNTs and LiOH·H_2_O were successfully synthesized, and the addition of Ni-CNTs resulted in the extensive dispersion of LiOH·H_2_O particles in the composite materials.

**Fig. 2:**
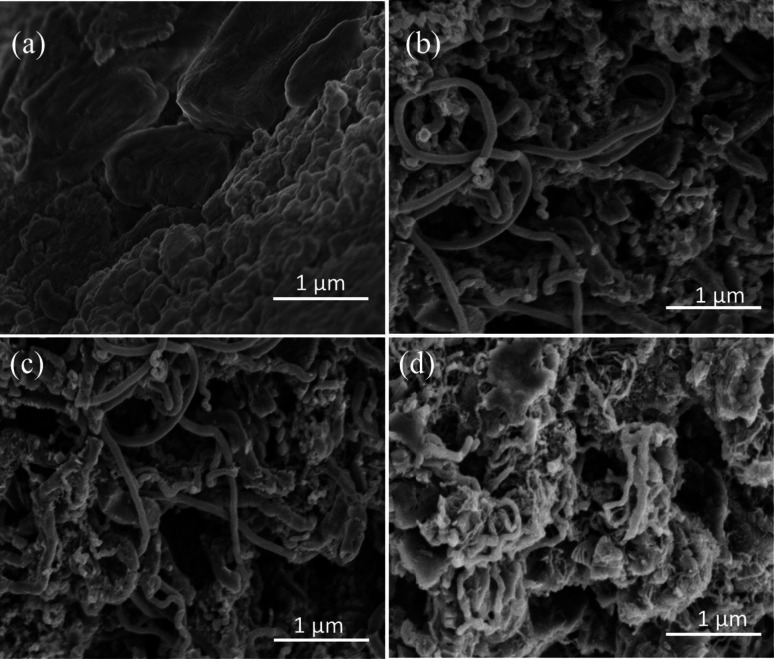
SEM images of (a) LiOH·H_2_O, (b) Ni-CNTs–LiOH·H_2_O-1, (c) Ni-CNTs–LiOH·H_2_O-2 and (d) Ni-CNTs–LiOH·H_2_O-3.

Fig. 2a–d provide the SEM images of LiOH·H_2_O, Ni-CNTs–LiOH·H_2_O-1, Ni-CNTs–LiOH·H_2_O-2, and Ni-CNTs–LiOH·H_2_O-3, respectively. As shown in Fig. 2a, before the addition of 3D-carbon nanoadditives, the bulk LiOH·H_2_O was aggregated with a large diameter (300 nm to 2 μm). After the modification of Ni-CNTs, no obvious change was observed in the regular structure for Ni-CNTs–LiOH·H_2_O-1 (Fig. 2b) and Ni-CNTs–LiOH·H_2_O-2 (Fig. 2c). This finding indicates that the intervention of LiOH·H_2_O did not lead to structural deterioration at a mass ratio < 23% and that LiOH·H_2_O was highly dispersed in the composite materials. This result agrees well with the XRD result. When the content of LiOH·H_2_O reached 39% (Ni-CNTs–LiOH·H_2_O-3), several LiOH·H_2_O particles could be seen on the surface of Ni-CNTs (Fig. 2d).

**Fig. 3:**
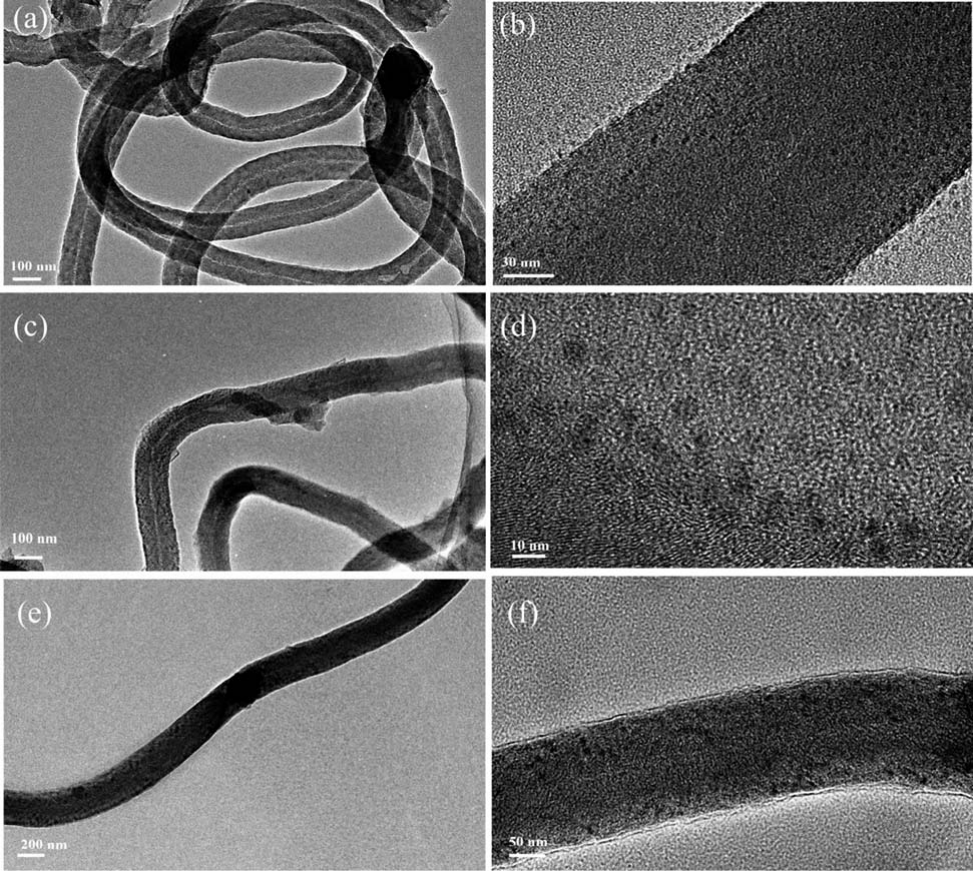
TEM images of (a) Ni-CNTs–LiOH·H_2_O-1, (c) Ni-CNTs–LiOH·H_2_O-2, (e) Ni-CNTs–LiOH·H_2_O-3, and HRTEM images of (b) Ni-CNTs–LiOH·H_2_O-1 (d) Ni-CNTs–LiOH·H_2_O-2 and (f) Ni-CNTs–LiOH·H_2_O-3.

Fig. 3 shows the TEM images of Ni-CNTs–LiOH·H_2_O with different contents of LiOH·H_2_O. In Fig. 3, LiOH·H_2_O nanoparticles with a diameter around 5 nm were successfully dispersed on Ni-CNTs (Fig. 3b) with clear particle structure. LiOH·H_2_O particle size showed a growth trend with increasing LiOH·H_2_O content in Ni-CNTs–LiOH·H_2_O composite materials (Fig. 3d and f). When LiOH·H_2_O content reached 39% (Fig. 3f), the LiOH·H_2_O nanoparticle size could increase to 15 nm, which is a bit bigger than the other two composite materials. Pure LiOH·H_2_O (Fig. 2a) existed in the form of stacked flakes and showed the largest particle size (300 nm to 2 μm). The addition of Ni-CNTs can effectively induce nanoscale LiOH·H_2_O dispersion, and the resulting particle size is markedly smaller than that of pure LiOH·H_2_O. During the synthesis of Ni-CNTs–LiOH·H_2_O composite materials, intermolecular interaction, such as hydrogen bonding, may exist between Ni-CNTs and LiOH·H_2_O owing to the presence of oxygen-containing functional groups, such as hydroxyl, carbonyl, and carboxyl groups,^38–40^ on the surface of Ni-CNTs. Therefore, proper additives supplied hydrogen bonding could show good ability for retarding the aggregation of LiOH·H_2_O. The porosity structures of LiOH·H_2_O were also measured by nitrogen adsorption–desorption. The BET specific surface area, pore volume, and average pore size are shown in Table 1. Ni-CNTs–LiOH·H_2_O samples show different textures. Owing to the addition of Ni-CNTs with different mass ratio, Ni-CNTs–LiOH·H_2_O shows a larger specific surface area than pure LiOH·H_2_O (15 m^2^ g^−1^). According to SEM and TEM characterization results, high specific surface area is an important cause of the nanoscale dispersion of LiOH·H_2_O particles.

**Table 1:** Texture parameters of carbon nanoadditives modified composed thermochemical materials and pure LiOH·H_2_O

Samples	Surface area (m^2^ g^−1^)	Pore volume (mL g^−1^)	Average pore size (nm)
Ni-CNTs	146	0.30	8.17
Ni-CNTs–LiOH·H_2_O-1	119	0.20	6.79
Ni-CNTs–LiOH·H_2_O-2	99	0.11	6.76
Ni-CNTs–LiOH·H_2_O-3	62	0.14	5.62
Pure LiOH·H_2_O	15	0.06	1.75

### 3.2. Heat storage performance test on LiOH·H_2_O-based TCMs

**Fig. 4:**
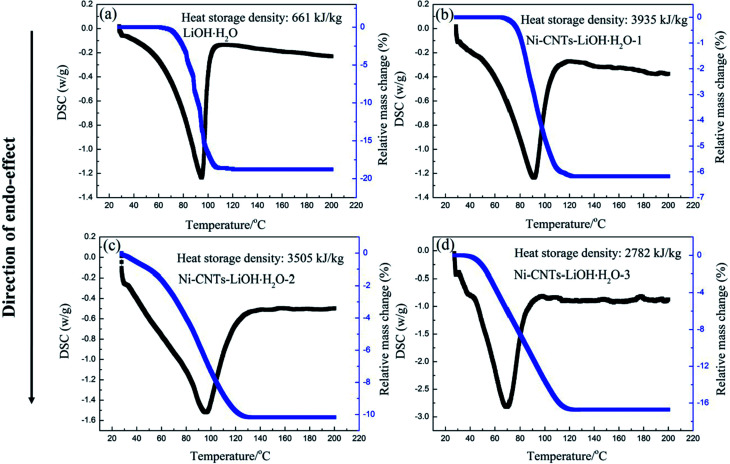
TG-DSC curves of as-synthesized samples: (a) pure LiOH after 1 h hydration, (b) Ni-CNTs–LiOH-1 after 1 h hydration, (c) Ni-CNTs–LiOH-2 after 1 h hydration, and (d) Ni-CNTs–LiOH-3 after 1 h hydration.

**Fig. 5:**
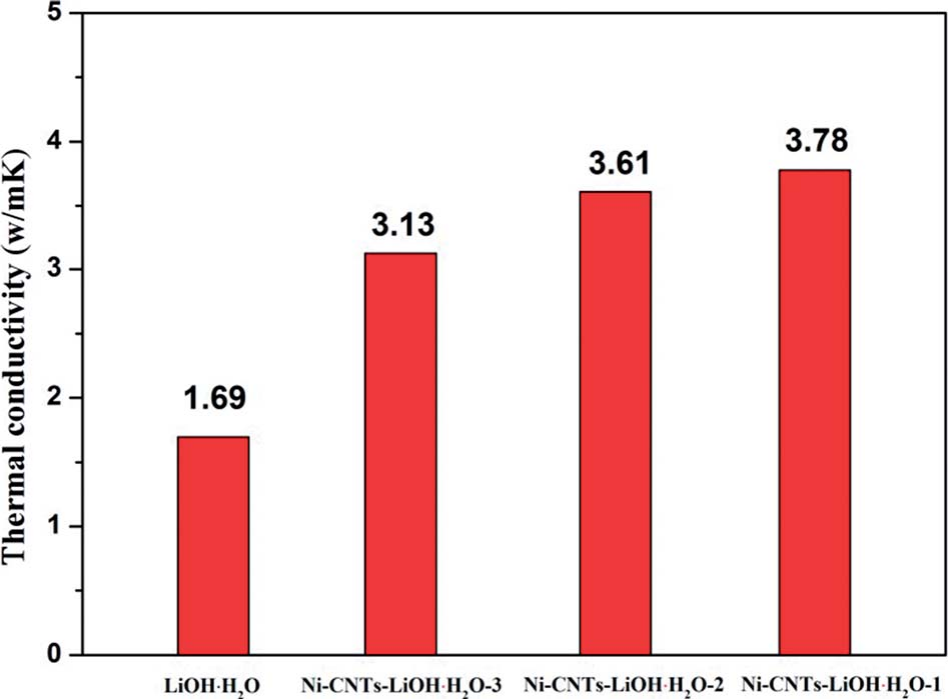
Thermal conductivity of LiOH·H_2_O, Ni-CNTs–LiOH·H_2_O-1, Ni-CNTs–LiOH·H_2_O-2 and Ni-CNTs–LiOH·H_2_O-3.

The heat storage performance test of pure LiOH·H_2_O, Ni-CNTs–LiOH·H_2_O-1, Ni-CNTs–LiOH·H_2_O-2 and Ni-CNTs–LiOH·H_2_O-3 were carried out and shown in Fig. 4. The *Y* axis is the amount of heating per unit time and mass (W g^−1^), and the *X* axis is temperature. The area of the curve is proportional to the change of enthalpy, as for heat storage materials it stands for the heat storage density. The pure LiOH·H_2_O heat storage density was only approximately 661 kJ kg^−1^ due to the slow reaction rate of LiOH and water vapor. In the hydration reaction, only about 42% of LiOH can be converted to LiOH·H_2_O after 1 h of hydration, as calculated by approximately 18% mass loss of H_2_O shown in Fig. 4a. By contrast Fig. 4b shows the DSC curve of 3D-carbon nanomaterial modified LiOH·H_2_O. After 1 h of hydration of the Ni-CNTs–LiOH·H_2_O composite, LiOH was fully hydrated to LiOH·H_2_O, and the heat storage density of Ni-CNTs–LiOH·H_2_O-1 normalized by LiOH·H_2_O content can reach 3935 kJ kg^−1^. High heat storage densities of 3505 kJ kg^−1^ and 2782 kJ kg^−1^ were observed in LiOH·H_2_O contained in Ni-CNTs–LiOH·H_2_O-2 (Fig. 4c) and Ni-CNTs–LiOH·H_2_O-3 (Fig. 4d), respectively. Therefore, compared with pure LiOH and with the same duration of the hydration reaction LiOH, and H_2_O molecule can be fully converted to LiOH·H_2_O owing to the introduction of Ni-CNTs, the hydration reaction rate of Ni-CNTs–LiOH·H_2_O was substantially improved. On the one hand, owing to the formation of hydrophilic functional groups on the surface of Ni-CNTs during preparation, H_2_O adsorption became easier and provided a completely different reaction interface between LiOH and water molecules. On the other hand, Ni-CNTs–LiOH·H_2_O showed ultrahigh heat storage density exceeding that of pure LiOH·H_2_O owing to the existence of hydrophilic functional groups^41^ and increased specific surface area, which markedly enhanced the dispersion of LiOH·H_2_O nanoparticles and the contact surface area with water molecules. The low specific surface area of pure LiOH·H_2_O may exert a negative effect on heat storage density. When the particle size reached nanoscale, the amount of surface atoms evidently increases; moreover, the crystalline field and binding energy of internal atoms were notably different from those of surface atoms, which possessed numerous dangling bonds owing to the lack of adjacent atoms. Hence, the unsaturated bonds in atoms show that nanoparticles present enhanced thermodynamic property.^42,43^ Meanwhile, due to increased number of surface atoms and the existence of hydrophilic functional groups, more H_2_O and LiOH can react, and therefore, heat storage performance can be improved. Furthermore, according to SEM and TEM characterization results, the heat storage density of Ni-CNTs–LiOH·H_2_O was higher than that of LiOH·H_2_O possible because of the smaller particle size of LiOH·H_2_O (5–15 nm) in Ni-CNTs–LiOH·H_2_O than that in pure LiOH·H_2_O (300 nm to 2 μm). Thus, small-sized nanoparticles can notably contribute to the enhancement of heat storage density for composite materials; as the particle size expands, the heat storage density of LiOH·H_2_O decreases. Furthermore, after the addition of Ni-CNTs to LiOH·H_2_O, the thermal conductivity of composite materials evidently increased and exceeded that of pure LiOH·H_2_O (Fig. 5) owing to the high thermal conductivity of Ni-CNTs. The highest thermal conductivity of Ni-CNTs–LiOH·H_2_O can reach 3.78 W m^−1^ K^−1^, which is 2.2 times higher than that of pure LiOH·H_2_O.

**Fig. 6:**
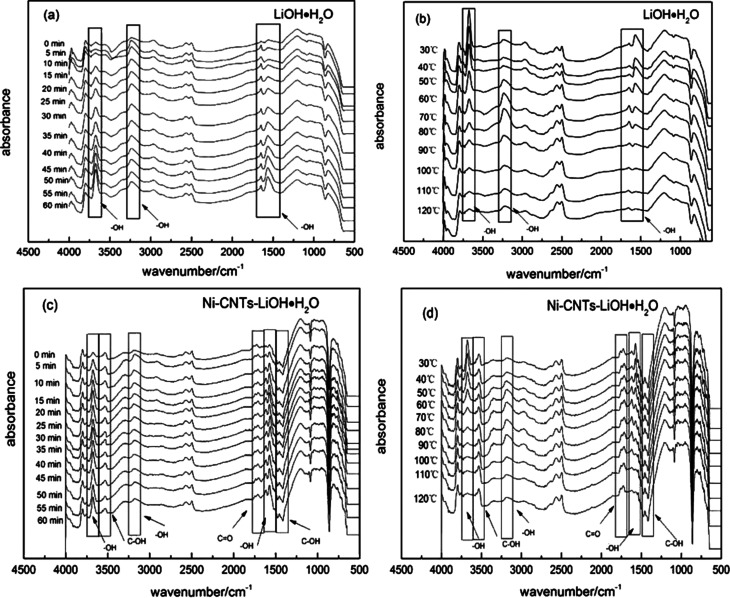
(a) The *in situ* DRIFT spectroscopy of the hydration reaction of LiOH; (b) the *in situ* DRIFT spectroscopy of dehydration reaction of LiOH·H_2_O; (c) the *in situ* DRIFT spectroscopy of hydration reaction of Ni-CNTs–LiOH; (d) the *in situ* DRIFT spectroscopy of dehydration reaction of Ni-CNTs–LiOH·H_2_O.

*In situ* DRIFT test was conducted to investigate the effect of hydrophilic functional groups of 3D-carbon nanotubes on the hydration/dehydration reaction of LiOH·H_2_O. Raw materials LiOH·H_2_O and the Ni-CNTs–LiOH·H_2_O composite were exposed to a flow of N_2_ (300 mL min^−1^) at 150 °C to decompose for 24 h. Then, the as-prepared samples LiOH and Ni-CNTs–LiOH were placed in the *in situ* reactor of an FT-IR spectrometer. The reactor was vacuumed and purged using a He flow. The *in situ* DRIFT test started with the hydration reaction of LiOH and H_2_O and finished with dehydration reaction of LiOH·H_2_O. Fig. 6a shows the *in situ* DRIFT spectroscopy of the hydration reaction of LiOH and H_2_O. The bands in the range of 3800–2200 cm^−1^ are always assigned to the stretching vibrations of structural OH groups and physical adsorbed H_2_O molecules.^44^ In the spectra, the peak at around 3679 cm^−1^ and 1573 cm^−1^ could be attributed to the stretching vibrations (*ν*_OH_) and bending vibrations (*β*_OH_) of the structure water in LiOH·H_2_O, respectively.^45^ Besides, broad peaks in the 2842–3423 cm^−1^ range were centered at around 3235 cm^−1^. This peak (3235 cm^−1^) and another peak at the lower band (1644 cm^−1^) were the *ν*_OH_ and *β*_OH_ of OH, respectively, in the physical adsorbed H_2_O.^44,46^ In Fig. 6a it could be also observed that when the hydration reaction was ready for start, the weak peaks, which stand for OH in structure H_2_O and physical adsorbed H_2_O molecules have been existed. This finding may be due to the reaction of LiOH and residual physical adsorbed H_2_O on the surface of LiOH. With prolonged hydration reaction, the peak intensities of structural OH (3679 cm^−1^ and 1573 cm^−1^) markedly increased at 15 min hydration. These peaks slowly increased during 15 min to 120 min hydration reaction, indicating the continuous reaction of LiOH and water steam and the decrease in hydration reaction rate. During this reaction, no obvious change could be observed for the peak intensities of OH (3235 cm^−1^ and 1644 cm^−1^) in physical adsorbed H_2_O because of the steady water steam flow in the *in situ* reactor. After the hydration reaction, the reactor was vacuumed and purged using a dry He flow, then LiOH·H_2_O was heated at a rate of 0.5 °C s^−1^ under the control of temperature-programmed technology.

Fig. 6b shows the *in situ* DRIFT spectroscopy of dehydration reaction of LiOH·H_2_O obtained from 1 h hydration of LiOH. When LiOH·H_2_O was heated to 60 °C, the peak intensities of OH in structural H_2_O (3679 cm^−1^ and 1573 cm^−1^) started to decrease. When the temperature exceeded 70 °C, the trend became more evident. Meanwhile, the peaks of OH (3235 cm^−1^ and 1644 cm^−1^) in physical adsorbed H_2_O appeared and increased from 60 °C to 80 °C, indicating the form of free water. The intensity of the peaks gradually decreased owing to easy desorption and blowing away of physical adsorbed water at elevated temperature.

Fig. 6c shows the *in situ* DRIFT spectroscopy of hydration reaction of Ni-CNTs–LiOH and H_2_O. During hydration reaction, the peak intensities of OH in structure H_2_O (3679 cm^−1^ and 1573 cm^−1^) showed a marked increase with 5 min hydration because of the existing hydrophilic groups, such as C–OH and C=O, on the surface of 3D-carbon nanotubes. After 20 min, the peaks were virtually unchanged, which indicating that the hydration reaction rate of LiOH and water steam was enhanced, exceeding that of pure LiOH. The peak intensities of OH (3181 cm^−1^ and 1625 cm^−1^) in physical adsorbed H_2_O did not notably change. The band at around 3520 cm^−1^ can be assigned to stretching vibrations, whereas the band at around 1400 cm^−1^ was attributed to the bending vibrations of C–OH.^44^ The band at 1720 cm^−1^ was assigned to C=O groups. When the water steam flowed into the reactor, the wavenumber of C=O groups shifted from 1720 cm^−1^ to 1710 cm^−1^ because of the formation of hydrogen bonding^2^ between C=O groups and adsorbed H_2_O. Owing to the effect of hydrogen bonding, the adsorption of H_2_O on the surface of Ni-CNTs–LiOH was considerably enhanced, facilitating an easier and more rapid reaction of H_2_O and LiOH than before.

Fig. 6d presented the *in situ* DRIFT spectroscopy of dehydration reaction of Ni-CNTs–LiOH·H_2_O. When Ni-CNTs–LiOH·H_2_O was heated to 50 °C, the peak intensities of OH in structural H_2_O (3679 cm^−1^ and 1573 cm^−1^) started to decrease; beyond 60 °C, LiOH·H_2_O was continuously dehydrated until the structure of H_2_O was fully lost. Meanwhile, the peaks of OH (3181 cm^−1^ and 1625 cm^−1^) in physical adsorbed H_2_O appeared, increased from 50 °C to 90 °C, and eventually decreased; this trend was ascribed to the easy removal of H_2_O at elevated temperature. The formed water could also produce hydrogen bonding with C=O groups (1720 cm^−1^) on the surface of Ni-CNTs, thereby causing a shift of the C=O band to a lower position (1710 cm^−1^). During the dehydration reaction from 30 °C to 120 °C, the intensities of C=O and C–OH (3520 cm^−1^ and 1400 cm^−1^) peaks showed no obviously change, indicating that the physical–chemical property of Ni-CNTs is steady within the total dehydration temperature range. The dehydration temperature of Ni-CNTs–LiOH·H_2_O was approximately 10 °C lower than that of pure LiOH·H_2_O; this finding is in good agreement with the results of the TG-DSC test.

**Fig. 7:**
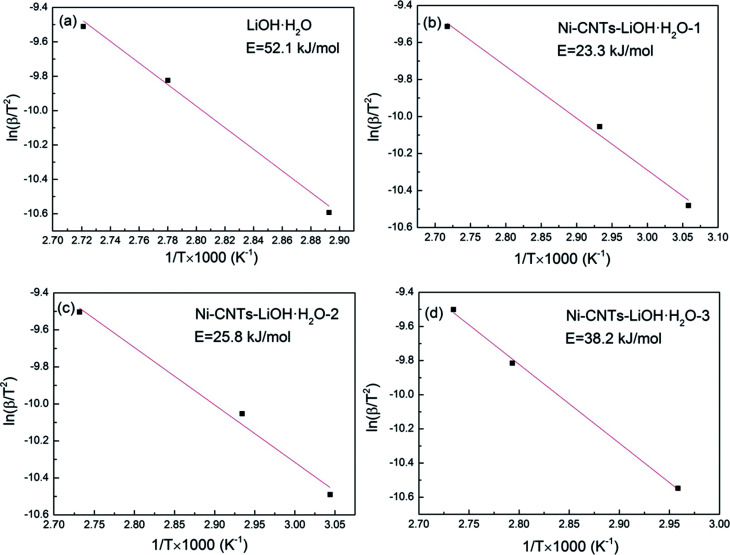
The activation energy of dehydration reaction of (a) LiOH·H_2_O, (b) Ni-CNTs–LiOH·H_2_O-1, (c) Ni-CNTs–LiOH·H_2_O-2, (d) Ni-CNTs–LiOH·H_2_O-3.

As shown in Fig. 7, the activation energies of the dehydration reaction of (a) LiOH·H_2_O, (b) Ni-CNTs–LiOH·H_2_O-1, (c) Ni-CNTs–LiOH·H_2_O-2, and (d) Ni-CNTs–LiOH·H_2_O-3 as obtained using the Ozawa method are 52.1, 23.3, 25.8, and 38.2 kJ mol^−1^, respectively.^36^ The activation energies of composite TCMs were markedly lower than that of pure LiOH·H_2_O due to the surface effect of nano-LiOH·H_2_O composite TCMs. The specific surface area or surface-to-volume ratio, which changes with particle size, depends on activation energy.^47^ By combining the SEM, TEM, and BET characterization results, it could be found that as the Ni-CNT content increased, the specific surface area of the composite TCMs also increased, whereas the particle size of LiOH·H_2_O decreased. The activation energy of LiOH·H_2_O dehydration reaction in composite TCMs showed similar trend to the particle size variation of LiOH·H_2_O. This trend can be attributed to diminished particle size, leading to increased surface-to-volume ratio and molar surface energy of nanoparticles, which mainly result in decreased activation energy.^48,49^ In summary, the addition of 3D-carbon nanomaterial Ni-CNTs can not only enhance water absorption at the LiOH particle surface but also decrease activation energy. Owing to the addition of Ni-CNTs, LiOH·H_2_O are dehydrated more easily, and the reaction mechanism of composited TCMs may deviate from that of pure LiOH·H_2_O because of the surface effect of 3D Ni-CNTs–LiOH·H_2_O nanocomposite materials during the thermochemical reaction process.

## 4. Conclusions

For investigating the effects of *in situ* formed 3D-carbon nanoadditives (Ni-CNTs) on the thermal performance of low-temperature LiOH·H_2_O-based composites as thermochemical heat storage materials, four kinds of LiOH·H_2_O-based composite TCMs were successfully constructed and characterized. Owing to the addition of 3D-carbon nanoadditives, the nanoscale (5–15 nm) LiOH·H_2_O particles were well dispersed in the composite with Ni-CNTs. The heat storage capacity and thermal conductivity of the composite materials were markedly improved. Meanwhile, the hydration rate was enhanced due to the hydrogen bonding formed between H_2_O and hydrophilic groups on the surface of Ni-CNTs, as shown by the combined results of *in situ* DRIFT spectroscopy characterization and the heat storage performance test. The activation energy for the thermochemical reaction process notably decreased after the addition of Ni-CNTs possibly because Ni-CNTs provide efficient hydrophilic reaction interface and exhibit surface effect in the hydration reaction. Among these TCMs, Ni-CNTs–LiOH·H_2_O-1 showed the lowest activation energy (23.3 kJ mol^−1^), highest thermal conductivity (3.78 W m^−1^ K^−1^), and highest heat storage density (3935 kJ kg^−1^), which is 5.9 times higher than pure lithium hydroxide after the same hydration duration. The heat storage density and the thermal conductivity of Ni-CNTs–LiOH·H_2_O are great higher than 1D MWCNTs and 2D graphene oxide modified LiOH·H_2_O. The selection of 3D carbon nanoadditives as composed part of the chemical heat storage materials is a very efficient way to enhance comprehensive performance of heat storage activity component.

## Conflicts of interest

There are no conflicts to declare.

## Supplementary Material
